# The Tumor Microbiome Reacts to Hypoxia and Can Influence Response to Radiation Treatment in Colorectal Cancer

**DOI:** 10.1158/2767-9764.CRC-23-0367

**Published:** 2024-07-10

**Authors:** Martin Benej, Rebecca Hoyd, McKenzie Kreamer, Caroline E. Wheeler, Dennis J. Grencewicz, Fouad Choueiry, Carlos H.F. Chan, Yousef Zakharia, Qin Ma, Rebecca D. Dodd, Cornelia M. Ulrich, Sheetal Hardikar, Michelle L. Churchman, Ahmad A. Tarhini, Lary A. Robinson, Eric A. Singer, Alexandra P. Ikeguchi, Martin D. McCarter, Gabriel Tinoco, Marium Husain, Ning Jin, Aik C. Tan, Afaf E.G. Osman, Islam Eljilany, Gregory Riedlinger, Bryan P. Schneider, Katarina Benejova, Martin Kery, Ioanna Papandreou, Jiangjiang Zhu, Nicholas Denko, Daniel Spakowicz, for the exORIEN Consortium

**Affiliations:** 1 Department of Radiation Oncology, The Ohio State University Comprehensive Cancer Center, Columbus, Ohio.; 2 Division of Medical Oncology, Department of Internal Medicine, The Ohio State University Comprehensive Cancer Center, Columbus, Ohio.; 3 Department of Health Sciences, The Ohio State University, Columbus, Ohio.; 4 Holden Comprehensive Cancer Center, University of Iowa, Iowa City, Iowa.; 5 Division of Oncology, Hematology and Blood & Marrow Transplantation, Holden Comprehensive Cancer Center, University of Iowa, Iowa City, Iowa.; 6 Department of Biomedical Informatics, The Ohio State University, Columbus, Ohio.; 7 Pelotonia Institute for Immuno-Oncology, The Ohio State University Comprehensive Cancer Center, Columbus, Ohio.; 8 Department of Internal Medicine, University of Iowa, Iowa City, Iowa.; 9 Department of Population Health Sciences, Huntsman Cancer Institute, University of Utah, Salt Lake City, Utah.; 10 Aster Insights, Hudson, Florida.; 11 Department of Cutaneous Oncology, H. Lee Moffitt Cancer Center and Research Institute, Tampa, Florida.; 12 Department of Immunology, H. Lee Moffitt Cancer Center and Research Institute, Tampa, Florida.; 13 Department of Thoracic Oncology, H. Lee Moffitt Cancer Center and Research Institute, Tampa, Florida.; 14 Department of Urologic Oncology, The Ohio State University Comprehensive Cancer Center, Columbus, Ohio.; 15 Department of Hematology/Oncology, Stephenson Cancer Center of University of Oklahoma, Oklahoma City, Oklahoma.; 16 Department of Surgery, University of Colorado School of Medicine, Aurora, Colorado.; 17 Department of Oncological Science, Huntsman Cancer Institute, University of Utah, Salt Lake City, Utah.; 18 Department of Biomedical Informatics, Huntsman Cancer Institute, University of Utah, Salt Lake City, Utah.; 19 Department of Internal Medicine, University of Utah, Salt Lake City, Utah.; 20 Clinical Science Lab, H. Lee Moffitt Cancer Center and Research Institute, Tampa, Florida.; 21 Department of Precision Medicine, Rutgers Cancer Institute of New Jersey, New Brunswick, New Jersey.; 22 Indiana University Simon Comprehensive Cancer Center, Indianapolis, Indiana.

## Abstract

Tumor hypoxia has been shown to predict poor patient outcomes in several cancer types, partially because it reduces radiation’s ability to kill cells. We hypothesized that some of the clinical effects of hypoxia could also be due to its impact on the tumor microbiome. Therefore, we examined the RNA sequencing data from the Oncology Research Information Exchange Network database of patients with colorectal cancer treated with radiotherapy. We identified microbial RNAs for each tumor and related them to the hypoxic gene expression scores calculated from host mRNA. Our analysis showed that the hypoxia expression score predicted poor patient outcomes and identified tumors enriched with certain microbes such as *Fusobacterium nucleatum*. The presence of other microbes, such as *Fusobacterium canifelinum*, predicted poor patient outcomes, suggesting a potential interaction between hypoxia, the microbiome, and radiation response. To experimentally investigate this concept, we implanted CT26 colorectal cancer cells into immune-competent BALB/c and immune-deficient athymic nude mice. After growth, in which tumors passively acquired microbes from the gastrointestinal tract, we harvested tumors, extracted nucleic acids, and sequenced host and microbial RNAs. We stratified tumors based on their hypoxia score and performed a metatranscriptomic analysis of microbial gene expression. In addition to hypoxia-tropic and -phobic microbial populations, analysis of microbial gene expression at the strain level showed expression differences based on the hypoxia score. Thus, hypoxia gene expression scores seem to associate with different microbial populations and elicit an adaptive transcriptional response in intratumoral microbes, potentially influencing clinical outcomes.

**Significance:**

Tumor hypoxia reduces radiotherapy efficacy. In this study, we explored whether some of the clinical effects of hypoxia could be due to interaction with the tumor microbiome. Hypoxic gene expression scores associated with certain microbes and elicited an adaptive transcriptional response in others that could contribute to poor clinical outcomes.

## Introduction

Colorectal cancer is the third most common and second most lethal cancer worldwide, accounting for 1.9 million cases and nearly 900,000 deaths every year (1). More than 60% of all colorectal cancer cases occur in the sigmoid colon and are diagnosed at stage II or above (2). The 5-year survival rate for colorectal cancer typically ranges from 90% for patients diagnosed with localized disease to 14% for those diagnosed with metastatic disease (3). Clinical management of colorectal cancer depends on the stage: surgery is the primary treatment option in the early stages, whereas neoadjuvant chemoradiotherapy (nCRT) followed by surgery is the standard of care for locally advanced stages (4). However, only about 15% of patients with colorectal cancer treated with nCRT achieve complete pathologic responses (5). Because dose escalation dramatically increases the risk of toxicity and exceeds the radiotolerance of adjacent normal tissues, understanding the mechanisms behind colorectal cancer resistance to nCRT therapy is of utmost clinical importance.

The human intestinal microbiome comprises 10^13^ to 10^14^ mutualistic microorganisms that play a crucial role in shaping the intestinal epithelium, harvesting nutrients, directing maturation of the host immune system, regulating defense against pathogens, and maintaining gut barrier function (6–10). Increasing lines of evidence suggest that dysbiosis or alteration of the intestinal microbiome composition and function is increasingly involved with the initiation and progression of colorectal cancer (11). Moreover, emerging studies suggest a direct association between intestinal microbiome dysbiosis and sensitivity to anticancer therapy (12–15). In particular, studies in gnotobiotic mice have shown that intestinal microbiota can shape the response to anticancer treatment, suggesting complex host–microbiome cross-talk (16, 17).

The microenvironment of the human gastrointestinal (GI) tract is generally hypoxic (physiologic hypoxia), showing steep oxygen gradients along the radial axis ranging from well-oxygenated subepithelial mucosa to the anaerobic lumen (18, 19). Many anaerobic microbes that thrive under anoxic conditions inhabit the lumen. Microbial colonization of colorectal cancer tumors by microbial taxa is, therefore, highly influenced by the route by which they reach the tumor and by the ability of the microorganism to survive the environmental oxygen within the tumor microenvironment (TME). Because hypoxia directly limits the efficiency of radiotherapy (20), we hypothesized that cross-talk between the colorectal cancer tumor microbiome and the hypoxic TME influences the tumor response to radiotherapy.

In this study, we analyzed RNA sequencing (RNA-seq) data from 141 pretreatment colorectal cancer samples from patients receiving radiotherapy to identify environmental hypoxia– and radiation treatment–dependent variations in the tumor microbiome. We then assessed these patterns for their impact on overall survival. We performed an *in vivo* validation of these concepts in heterotopic model CT26 tumors to identify microbiome composition and gene expression as a function of tumor oxygenation. A comparison of host animals from BALB/c and athymic nude strains identified hypoxia-dependent microbial populations and adaptive gene-level responses to varying oxygen conditions.

## Methods

### Study design

The Oncology Research Information Exchange Network (ORIEN) is an alliance of 18 US cancer centers established in 2014. All ORIEN alliance members utilize a standard Total Cancer Care^®^ (TCC) protocol. As part of the TCC study, participants agree to have their clinical data followed over time, undergo germline and tumor sequencing, and be contacted by their provider if an appropriate clinical trial or other study becomes available (21). TCC is a prospective cohort study with a subset of patients enrolled in the ORIEN Avatar program, including research-use-only–grade whole-exome tumor sequencing, RNA-seq, germline sequencing, and deep longitudinal clinical data collection with lifetime follow-up. Nationally, more than 325,000 participants have enrolled in TCC. Aster Insights, ORIEN’s commercial and operational partner, harmonizes all abstracted clinical data elements and molecular sequencing files into a standardized, structured format to enable aggregation of de-identified data for sharing across the network. This study included 2,755 ORIEN Avatar patients diagnosed with melanoma, sarcoma, or thyroid, pancreatic, colorectal, or lung cancer who consented to the TCC protocol from the participating members of ORIEN. Of these, 500 patients had colon adenocarcinoma, and 95 had rectal adenocarcinoma (Table 1).

**Table 1:** Demographic information for the ORIEN patients with COAD or rectum adenocarcinoma (READ) tumors, stratified by radiation treatment

	No	Unknown	Yes	*P*
*n*	355	57	141	
Sex = male (%)	180 (50.7)	28 (49.1)	87 (61.7)	0.069
Age [mean (SD)]	60.73 (12.88)	59.05 (10.20)	57.79 (11.67)	0.051
BMI [mean (SD)]	28.23 (6.73)	32.11 (11.23)	27.40 (6.50)	0.038
Stage (%)				<0.001
Unknown	218 (61.4)	36 (63.2)	38 (27.0)	
0	3 (0.8)	0 (0.0)	0 (0.0)	
I	15 (4.2)	2 (3.5)	11 (7.8)	
II	13 (3.7)	4 (7.0)	16 (11.3)	
III	19 (5.4)	5 (8.8)	31 (22.0)	
IV	87 (24.5)	10 (17.5)	45 (31.9)	
Race (%)				0.167
Black	24 (6.8)	3 (5.3)	9 (6.4)	
Other	26 (7.3)	10 (17.5)	14 (9.9)	
White	305 (85.9)	44 (77.2)	118 (83.7)	
Cancer = READ (%)	18 (5.1)	11 (19.3)	59 (41.8)	<0.001
Buffa hypoxia score [mean (SD)]	7.70 (23.33)	10.56 (21.68)	4.28 (25.47)	0.182
Hypoxia status: median = low (%)	165 (46.5)	27 (47.4)	67 (47.5)	0.975
Hypoxia status: tertiles (%)				0.987
High	125 (35.2)	19 (33.3)	48 (34.0)	
Low	118 (33.2)	19 (33.3)	45 (31.9)	
Medium	112 (31.5)	19 (33.3)	48 (34.0)	
Vital status = dead (%)	82 (23.1)	9 (15.8)	33 (23.4)	0.446

### Sequencing methods

ORIEN Avatar specimens underwent nucleic acid extraction and sequencing at HudsonAlpha or Fulgent Genetics. Frozen and optimal cutting temperature tissue RNA extraction was performed using the Qiagen RNeasy plus mini kit, generating a 216 bp average insert size. For formalin-fixed, paraffin-embedded tissue, the Covaris Ultrasonication FFPE DNA/RNA kit was utilized to extract DNA and RNA, respectively, generating a 165 bp average insert size. RNA-seq was performed using the Illumina TruSeq RNA Exome with single library hybridization, cDNA synthesis, library preparation, and sequencing (100-bp paired reads at HudsonAlpha; 150-bp paired reads at Fulgent) to a coverage of 100 M total reads/50 M paired reads. RNA-seq tumor pipeline analysis was processed according to the workflow outlined in the following section using GRCh38/hg38 human genome reference sequencing and GenCode build version 32. Adapter sequences were trimmed from the raw tumor sequencing FASTQ file. Adapter trimming via k-mer matching was performed along with quality trimming and filtering, contaminant filtering, sequence masking, guanine–cytosine filtering, length filtering, and entropy filtering. The trimmed FASTQ file was used as input to the read alignment process. The tumor adapter–trimmed FASTQ file was aligned to the human genome reference (GRCh38/hg38) and the GENCODE genome annotation v32 using the STAR aligner. The STAR aligner generates multiple output files for gene fusion prediction and expression analysis. RNA expression values were calculated and reported using estimated mapped reads, fragments per kilobase of transcript per million mapped reads, and transcripts per million mapped reads at both the transcript and gene levels based on transcriptome alignment generated using STAR. We used PowerFecal pro kits from Qiagen to extract RNA and DNA for model murine tumors.

### {exotic} processing

Microbes were classified in human and mouse samples using the {exotic} pipeline. This entails removing as many human or mouse reads as possible by aligning to the appropriate genome using STAR. The hg38 genome was used for human samples, the GRCm39 genome was used for mouse samples, and annotated genes were further used in gene expression analyses. Reads left unclassified after STAR alignment were then aligned to a Kraken 2 database customized to include fungi and archaea. Bayesian re-estimation of abundance with Kraken was used to determine the likely species of all classified reads. The R package {exotic} (22) was then used to decontaminate the results and to normalize the human samples to account for processing at different sequencing centers.

### Hypoxia score generation

Hypoxia scores were generated using the R package {tmesig} (23) for Buffa, Winter, and Leonard signatures. The R package {mt.surv} was used to compare these scores’ relationships with overall survival, and Buffa (24) was chosen to represent hypoxia in further analyses. For all analyses comparing low and high hypoxia scores, samples were defined as having low hypoxia if their hypoxia score was in the lower tertile of the data and high hypoxia if their score was in the upper tertile.

### Time-to-event analyses

Kaplan–Meier survival curves were generated, and Cox proportional hazards models were applied using the R package {survival} and visualized using {ggsurv}.

### Differential abundance and expression analyses

The R package {DESeq2} was used to compare the abundance of microbes in low- and high-hypoxia tumors for both The Cancer Genome Atlas (TCGA) and ORIEN data. It was also used to compare microbe abundances in low- and high-hypoxia tumors in the mouse experiments and the behavior of microbe gene expressions in high- and low-hypoxia mouse tumors. Demographic information for this population was similar to the ORIEN dataset (Table 2).

**Table 2:** Demographic information for the COAD and READ samples in TCGA database, comparing demographic information between COAD and READ

	COAD	READ	*P*
*N*	478	166	
Sex = Male (%)	252 (52.9)	90 (54.5)	0.791
Age [mean (SD)]	67.58 (13.01)	65.06 (11.67)	0.028
BMI [mean (SD)]	29.46 (16.55)	27.12 (5.81)	0.24
Stage (%)			0.199
I	80 (16.7)	30 (18.1)	
II	186 (38.9)	51 (30.7)	
III	132 (27.6)	51 (30.7)	
IV	66 (13.8)	24 (14.5)	
Unknown	14 (2.9)	10 (6.0)	
Race (%)			0.002
Black	62 (13.0)	6 (3.6)	
Other	185 (38.7)	80 (48.2)	
White	231 (48.3)	80 (48.2)	
Buffa hypoxia score [mean (SD)]	15.46 (13.84)	12.15 (13.67)	0.008
Hypoxia status (%)			NaN
High	0 (NaN)	32 (37.2)	
Low	0 (NaN)	30 (34.9)	
Medium	0 (NaN)	24 (27.9)	
Vital status = dead (%)	103 (21.6)	27 (16.4)	0.18

The ORIEN dataset, with which this is compared, contains a larger fraction of READ samples.

### Amplicon-based (16S) sequencing of human tumor samples

The bacterial 16S rRNA gene was amplified from fresh frozen tumor (*n* = 31) and adjacent normal (*n* = 31) tissues from 31 patients. Microbial and tumor cells were lysed on a PowerLyzer 24 at 2,000 rpm for 30 seconds, and then DNA was purified using an AllPrep mini kit (Qiagen). The bacterial rDNA was amplified using V3 to V4 primers and KAPA HiFi enzyme (50°C, 30 seconds; 72°C, 2 × 20 cycles). Magnetic beads cleaned amplicons, and sequencing libraries were generated using a QIAseq kit (Qiagen) following the manufacturer’s instructions. Libraries were sequenced on a MiSeq 2 × 300 (600 cycles) using a V3 reagent kit (Illumina). Demultiplexed FASTQs were filtered for quality and length (340–440 bp), and processed to amplicon sequence variant using {dada2}. Taxonomy was assigned with {exotic} using the same database as for the tumor RNA-seq.

### Athymic nude and BALB/c mice tumors

MC38 (RRID: CVCL_B288) and CT26 cells (RRID: CVCL_7254) were obtained from ATCC. Female 7-week-old athymic nude (RRID: IMSR_CRL:088) and BALB/c (RRID: IMSR_APB:4790) mice were purchased from Charles River (housed in groups of five) and were injected subcutaneously into the right flank with either 1 million MC38 or 0.5 million CT26 cells with tumor growth measured using calipers using the formula (*L* × *W*^2^)/2. Radiotherapy was delivered by a single tangential beam from the small animal radiation research platform. Untreated tumors were typically harvested on day 16 after inoculation when most tumors were at removal criteria. Mice stool was collected before tumor harvesting. All animal experiments were done in accordance with the approved Ohio State University Institutional Animal Care and Use Committee protocol (protocol # 2012A00000124-R3). Cells were grown in DMEM containing 25 mmol/L D-glucose, 4 mmol/L glutamine, and 44 mmol/L sodium bicarbonate in 5% CO_2_. All cell lines have been authenticated using STR profiling within the past 3 years. All experiments were performed with *Mycoplasma*-free cells, as determined by routine PCR testing in 3 to 4-month intervals.

### Microbial culture and healthy gut mixture

Representative bacterial strains *Akkermansia muciniphila* (ATCC^®^ BAA-835), *Lactobacillus acidophilus* (ATCC^®^ 4356), *Streptococcus thermophilus* (ATCC^®^ 19258), *Bacteroides ovatus* (ATCC^®^ 8483), and *Dorea formicigenerans* (ATCC^®^ 27755) were obtained from ATCC. Fecal bacteria isolates were collected from three healthy adult volunteers as described previously (protocol approval was obtained from the Institutional Review Board, and written informed consent was documented for each individual; ref. 25). All bacterial strains were maintained under standard culture conditions as previously described (26). Briefly, overnight bacterial cultures were established in Gifu anaerobic medium (GAM; HiMedia). The cultures were maintained in an anaerobic environment at 37°C using a Coy Laboratory anaerobic chamber (Coy Lab). To estimate growth, the final optical density of bacterial cultures was measured using an ELx808 absorbance plate reader (BioTek). Drop plate analysis was also used to quantify the colony-forming units in each culture. Before injections, bacterial cultures were centrifuged and then resuspended at a final concentration of 3 × 10^9^ colony-forming units per injection.

### Mouse sequencing processing: Microbe genes

RNA-seq data from the mouse tumors were classified using HUMAnN 3.0 with MetaPhlAn and the default ChocoPhlAn database to obtain information on the gene expression of microbes in the mouse experiments.

### Data availability

The Ohio State University Institutional Review Board approved data access in an Honest Broker protocol (2015H0185) and TCC protocol (2013H0199) in coordination with Aster Insights. FASTQs for mouse RNA-seq are available in two batches published to Zenodo at 10.5281/zenodo.11211908 and https://doi.org/10.5281/zenodo.11212525. Analysis scripts and data to regenerate all figures and tables are available at: https://github.com/spakowiczlab/exorien-recrad.

## Results

### Tumor hypoxia and survival after radiotherapy

**Figure 1:**
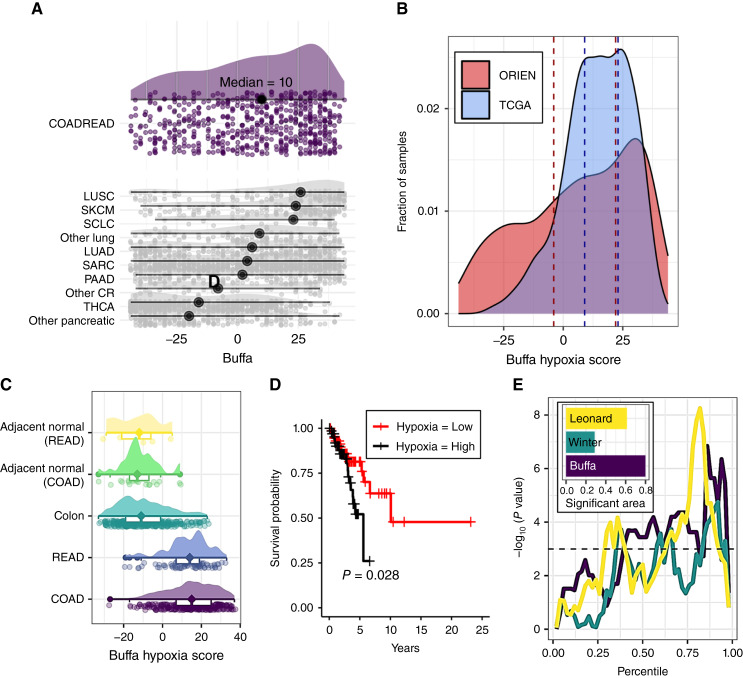
Hypoxia exists in colorectal cancer and predicts poor patient outcomes after X-ray therapy. **A,** Hypoxia expression signature scores for the ORIEN dataset across tumor sites showing a wide distribution for COAD and READ samples. The median values are indicated with a black circle. Abbreviated cancer names are lung squamous cell carcinoma (LUSC), skin cutaneous melanoma (SKCM), small cell lung cancer (SCLC), lung adenocarcinoma (LUAD), sarcoma (SARC), pancreatic adenocarcinoma (PAAD), other colorectal cancers (Other CR), and thyroid carcinoma (THCA). **B,** A comparison of the Buffa score histogram of ORIEN with TCGA colorectal cancer datasets shows a similar mode in both but with a wider distribution for ORIEN. Tertiles used to designate “high” and “low” hypoxia are marked with dashed lines. **C,** Comparison of Buffa scores in normal adjacent and normal GTEx data compared with colon and rectal cancers (TCGA). **D,** For ORIEN patients receiving radiotherapy, a Kaplan–Meier curve stratifying the patient population into upper and lower hypoxia tertiles shows that hypoxic tumors have a worse patient outcome. **E,** Cox proportional hazard *P* values calculated by stratifying the hypoxia scores at every percentile (*x*-axis). The larger area above the horizontal dotted line indicates the strong separation of survival at a range of threshold values and is therefore more robust than single threshold analyses for small sample sizes. The total area of the Buffa hypoxia score was slightly larger than the others (inset).

Virtually all solid tumor types are characterized by some level of hypoxia. One way to approximate tumor hypoxia is to use quantitative gene expression signatures of hypoxia-regulated genes. For Fig. 1A , we calculated the values and distribution of the hypoxia expression score (24) across cancer types within the ORIEN dataset (24). This hypoxia signature is similar to others and scores sets of genes induced in response to hypoxia primarily by the hypoxia-inducible factor 1 (HIF1) transcription factor. This gene family can be extracted from tumor mRNA data in publicly available clinical datasets to identify tumors with significant levels of hypoxia. In colorectal cancers, we did not find a significant association between hypoxia and patient characteristics such as tumor stage or sex (Supplementary Fig. S1). We also see no differences in hypoxia correlating with age, gender, body mass index (BMI), stage, race, or when subdividing rectal adenocarcinoma versus colon adenocarcinoma (Table 1).

We processed RNA-seq data from 141 patients with colorectal cancer available through the ORIEN network and calculated hypoxia scores (24) for each sample. Colorectal adenocarcinoma (COADREAD) ranks among the more hypoxic cancer types with a median hypoxia score of 10 (Fig. 1A). Comparing cancers from different sites, the distribution of hypoxia scores ranges from lowest in thyroid cancer (median hypoxia expression score of −16) to highest in lung squamous cell cancer (median hypoxia expression score of 26). We then cross-validated the ORIEN COADREAD hypoxia scores by comparing them with the publicly available TCGA COADREAD dataset. Demographic information for TCGA patients is similar to that of the ORIEN patients and is available in Table 2. We found substantial overlap in the distribution of scores between the independent patient datasets, confirming the robustness of this analysis and indicating that the many COADREAD samples were distributed within the high-end hypoxia expression score region (Fig. 1B). When we compare the average hypoxia scores in the normal colon [from the Genotype-Tissue Expression (GTEx) database of normal tissues] with the scores in the adjacent normal colon or rectum in TCGA database with the colon or rectal cancer, we can see a significant increase in the cancerous samples, and very similar distribution in the colon and rectal cancers (Fig. 1C). Current molecular models of radiation-induced DNA damage specify that oxygen is needed for the “fixation” of damage; thus, hypoxia directly impairs the efficiency of radiotherapy (27). Therefore, we tested whether elevated hypoxia scores would correlate with poor survival of patients with colorectal cancer treated with radiotherapy. We stratified ORIEN patients who underwent radiotherapy at the upper and lower tertiles of the hypoxia expression score, and a Cox proportional hazards model shows that a high hypoxic score significantly correlates with poor overall patient survival (*P* = 0.028; Fig. 1D; numbers at risk in Supplementary Table S1). Demographic variables were not significantly different between these groups (Supplementary Table S2). To ensure that the association with survival was robust to where we selected the “high” versus “low” stratification and to identify the best expression signature for hypoxia for this dataset, we calculated the hypoxia scores for three published signatures (3–5) and then stratified the data at all possible thresholds as a percentile of the distribution (Fig. 1E). The hypoxia expression score by Buffa and colleagues (24) significantly associated with survival over a wide range of stratification thresholds. By testing all possible thresholds, we created a curve and then integrated the area of *P* values below a 0.05 threshold. The hypoxia score from Buffa and colleagues showed the greatest area and was used for all further analyses (Fig. 1E, inset).

### Association between hypoxia and tumor microbes

**Figure 2:**
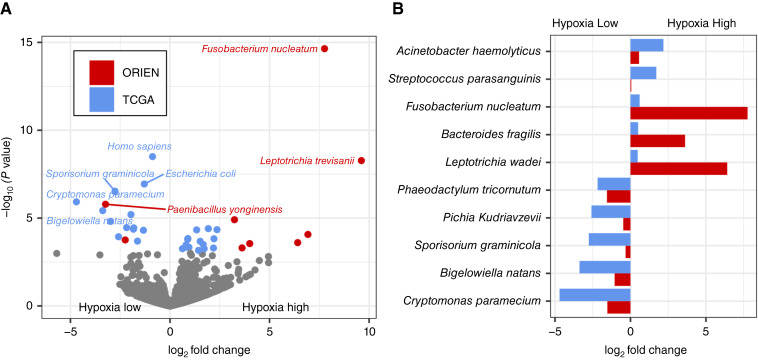
Hypoxia score associated with significant differences in microbial populations in colorectal cancer. **A,** Volcano plot of microbes from ORIEN and TCGA tumors showing strain differences when tumors are stratified by high vs. low hypoxia scores (tertiles). **B,** Fold change of microbes that show hypoxic enrichment in both ORIEN and TCGA datasets. XRT, X-ray therapy.

Significant evidence suggests that the composition of the gut microbiome correlates with the initiation and progression of colorectal cancer and affects tumor sensitivity to anticancer therapy (15). Accordingly, we evaluated tumor microbiota composition in ORIEN and TCGA patient samples using the exogenous sequences in tumors and immune cells {exotic} tool for quantifying microbe abundances in tumor RNA-seq data (22). To identify taxa associated with high levels of tumor hypoxia, we stratified patient samples into tertiles of increasing hypoxia scores (24) and quantified log_2_ abundance fold change between the bottom and top ranked tertiles (Fig. 2A). This analysis was performed on both ORIEN and TCGA colorectal cancer datasets, and several microbes were concordant in both datasets (significant and in the same direction; Fig. 2B; all fold changes are available in Supplementary Table S3). Interestingly, *Fusobacterium nucleatum* has been associated with poor outcomes in patients with colorectal cancer (15, 16) and is elevated in hypoxic tumors (Fig. 2A and B). Hypoxic tumors also contained significantly more M0-type macrophages, resting NK cells, memory B cells, CD4 T cells (regulatory T cells and resting memory), and activated dendritic cells, estimated by a deconvolution approach (Supplementary Fig. S2; Supplementary Table S4). Amplicon-based sequencing of a subset of the ORIEN samples confirmed the presence of *Fusobacterium*, which had a higher median abundance in hypoxic tumors, though the difference between the distributions was not statistically significant (Supplementary Fig. S3).

### Association between tumor microbes and survival after radiotherapy

**Figure 3:**
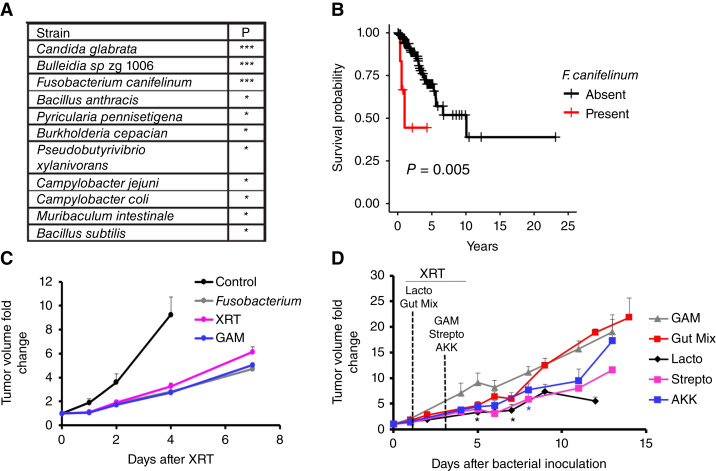
The presence of certain microbes in ORIEN tumors correlates with worse outcomes after radiotherapy. **A,** List of strains whose interactions with hypoxia in the tumor are significantly associated with survival outcomes in ORIEN patients who received radiation treatment. **B,** Kaplan–Meier survival curve showing an association between the presence of *F. canifelinum* and patient outcome. A risk table for this result is available as Supplementary Table S6. **C,** Model CT26 tumors grown in immune-deficient mice and inoculated with *Fusobacterium* show no increase in tumor growth delay after radiotherapy. **D,** Similarly, model tumors inoculated with several other bacterial strains show significant increases in tumor growth delay after radiotherapy. The strains included are *Lactobacillus* (Lacto), a mixed population from a healthy donor (Gut Mix), bacterial growth medium (GAM), *Streptococcus* (Strepto), and *Akkermansia* (AKK). Error bars are ±SEM. *P* values were calculated against GAM using the *t* test. *, *P* < 0.05.

We next analyzed the intratumoral microbial populations in these patients for strains that correlated with the outcome of radiotherapy for colorectal cancer. We identified 11 microbes in which presence correlated with significantly worse outcomes after radiotherapy (Fig. 3A; complete results in Supplementary Table S5). Strains showing the strongest statistical correlation included *Candida glabrata*, a fungus, and *Fusobacterium canifelinum* and *Bulleidia* sp. zg 1006, both bacteria. *F*. *canifelinum* is an anaerobic, Gram-negative bacilli that is not generally present in a healthy human gut microbiome but typically occurs in the oral cavity (28). The closely related *F. nucleatum* has been identified in recurrent colorectal cancer, and the presence of the *Fusobacterium* strain promotes colorectal cancer development (29, 30) and chemoresistance (16). We, therefore, asked whether the combined presence of hypoxia and *F. canifelinum* affected treatment outcomes in patients with colorectal cancer undergoing radiotherapy. Analysis of patients with colorectal cancer treated with radiotherapy identified that the presence of *F. canifelinum* or a high hypoxia score (24) is significantly associated with reduced overall survival (*P* value *F. canifelinum* = 0.005; Fig. 3B; numbers at risk in Supplementary Table S6).

To experimentally validate our bioinformatic observations *in vivo*, athymic nude mice were inoculated heterotopically with the mouse colorectal cancer line MC38. Upon reaching 100 mm^3^, MC38 tumor–bearing mice were randomized into groups receiving either nothing as control, intratumoral bacterial growth medium (GAM), or cultured *F. nucleatum* grown in GAM to achieve a ratio of 1:1 bacteria to tumor cells. The 1:1 ratio was chosen after control experiments indicated it as a dose that did not elicit an acute inflammatory response. After 48 hours, mice were either sham irradiated or given one dose of 8 Gy external beam radiation using the small animal radiation research platform. We observed no significant change in tumor response to therapy with the introduction of *F. nucleatum* (Fig. 3C).

We, therefore, decided to test other bacterial strains for effects on radiation response and repeated this experiment using several common gut bacterial strains: (i) microbial coculture representative of a healthy intestinal microbiome (gut mix); (ii) *Lactobacillus* spp.; (iii) *Streptococcus* spp.; or (iv) *Akkermansia* spp., also by intratumoral injection at a 1:1 ratio of microbe to tumor cell, followed 48 to 72 hours later by an 8-Gy dose of local radiation. Figure 3D shows that the injection of *Lactobacillus* or *Streptococcus* bacterial strains into heterotopic colorectal cancer tumors resulted in a pronounced increase in tumor growth delay after radiation. Taken together, these findings support the idea that intratumoral microbes can influence the response of model tumors to radiotherapy in a strain-dependent manner.

### The role of hypoxia and the immune system in tumor colonization by microbes

**Figure 4:**
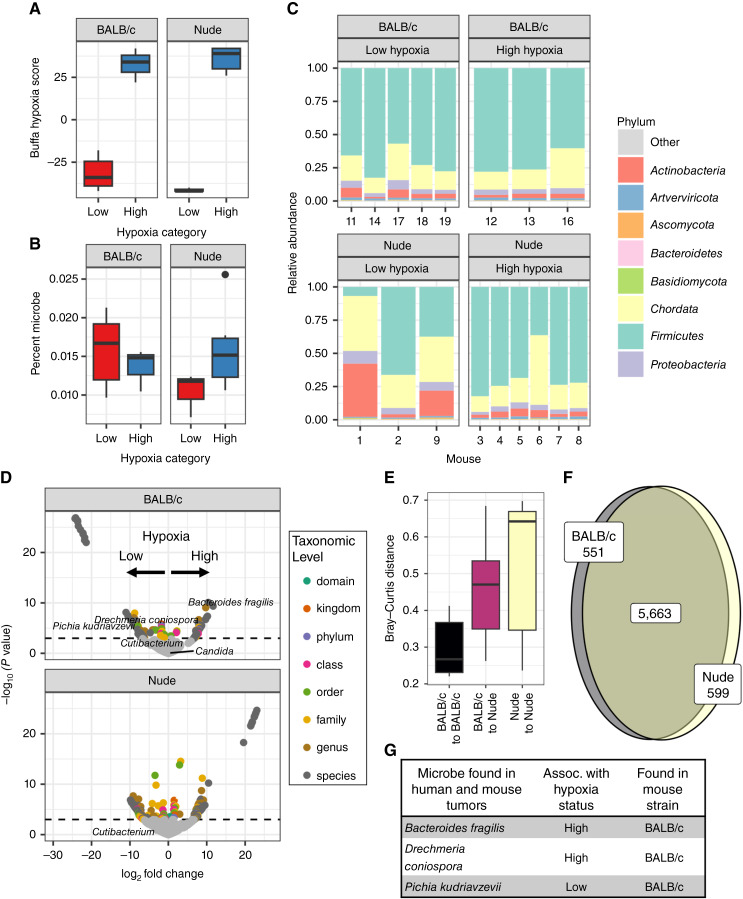
Model tumors also have oxygen-dependent microbial strain differences. **A,** CT26 colorectal cancer tumors grown in immune-deficient hosts have increased microbial burden with increased hypoxia. **B,** CT26 colorectal cancer tumors grown in immune-deficient or immune-competent hosts can be stratified into low and high hypoxia groups by the tumor Buffa score. **C,** Metatranscriptomic identification of microbial strains spontaneously colonizing individual tumors. **D,** Volcano plot of strains identified in (**C**) with tropism for tumor of either high or low hypoxia score. **E,** Bray–Curtis distances of gut samples from nude and BALB/c mice, showing comparisons within each group and with each other. **F,** Venn diagram of species shared between nude and BALB/c mouse gut microbiomes. **G,** Enumeration of the consistencies between the mouse and human data. We show microbes enriched in hypoxic or normoxic tumors in humans and the particular mouse strain noted in the table.

We next investigated the impact of a hypoxic TME on spontaneous tumor microbial colonization in model tumors in mice. We inoculated a cohort of 10 BALB/c and 10 athymic nude host mice with the CT26 murine colorectal cancer cell line. The mice received 0.5 × 10^6^ CT26 cells subcutaneously in the flank, and their tumors were harvested upon reaching 500 mm^3^ for nucleic acid extraction. Hypoxia scores (24) were calculated from tumor RNA for each sample, and the tumors were stratified at the mean hypoxia score (24) into normoxic (low hypoxia expression score) and hypoxic (high hypoxia expression score) tumors in each mouse host (Fig. 4A). We did not detect strong differences in estimated immune populations across both datasets (Supplementary Fig. S4; Supplementary Table S7), though deconvolution predicted differences in the number of tumor-infiltrating lymphocytes in the nude mice (Supplementary Table S8). We used metatranscriptomic sequencing of exogenous RNA to identify microbial strains enriched in hypoxic tumors. In the immune-competent model, the bacterial burden was equivalent in both low- and high-hypoxia tumors. However, in the tumors grown in nude mice, the burden was higher in hypoxic tumors (Fig. 4B). Nude mice showed a larger fraction of anaerobic strains in the *Firmicutes* phylum (Fig. 4C). Microbes that were significantly enriched in the normoxic and hypoxic tumors for each host were identified (Fig. 4D; complete results in Supplementary Table S9). To better understand the differences in the two host strains of mice, we collected stool samples for analysis of the gut microbiome by 16S amplicon sequencing. A comparison of individuals in this experiment by Bray–Curtis distance revealed that the immune-competent host samples were most similar, whereas the immune-deficient hosts were highly variable (Fig. 4E). However, very similar taxa were observed in all mice; approximately 80% of the 16S sequences were common to both hosts, whereas 10% were unique to each of the BALB/c and athymic nude host animals (Fig. 4F). Interestingly, there was an overlap of three of the strains found preferentially in the hypoxic tumors in both humans and mice (Fig. 4G; complete overlap between human and mouse data in Supplementary Table S10). The amount of *Pichia* significantly correlated with the estimated number of tumor monocytes (Kruskal–Wallis, FDR-corrected *P* value 0.03; all correlation results in Supplementary Table S11).

### *Cutibacterium* alters its transcription in hypoxic tumors

**Figure 5:**
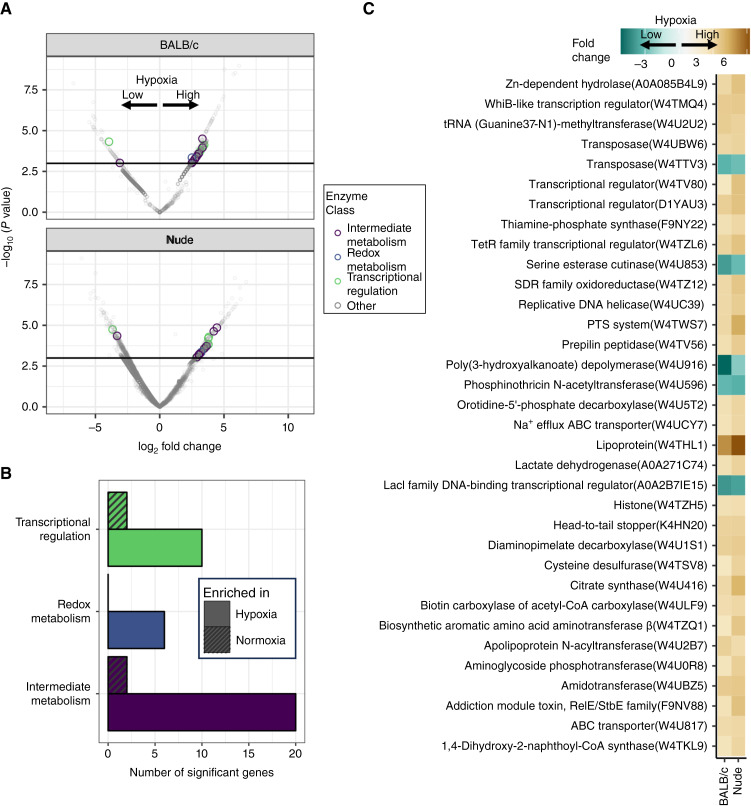
Oxygen-responsive gene expression of *Cutibacterium* within model tumors. **A,** Volcano plot of metatranscriptomic analysis of transcripts from *Cutibacterium* found in low or high hypoxia score CT26 tumors grown in either immune-deficient or immune-competent host animals (1,295 total transcripts identified). **B,** Bar plot showing the total number of significantly enriched genes in the hypoxic and normoxic conditions enriched in each enzyme class as notated in (**A**). **C,** Table of the 34 annotated transcripts that showed significant concordant changes to hypoxia score in both datasets (48 total, 39 pos, and 9 neg).

However, the most consistent colonization of all tumors was observed with the genus *Cutibacterium* from the phylum *Actinobacteria* (Fig. 4D). *Cutibacterium* is a Gram-positive anaerobic bacillus typically found in the skin and associated with acne vulgaris; however, emerging studies identified a possible role for the microbe in prostate cancer immunosuppression (31). Based on the prevalence of *Cutibacterium* in all analyzed samples, we performed transcriptomic analysis of this species in both the BALB/c and nude mouse hosts. We analyzed the mechanisms by which the same microbial strain responds to this TME variable (tumor oxygenation as determined by the hypoxia expression score) in different mouse hosts. Differential microbial RNA analysis revealed significant differences between microbial gene expression in normoxic versus hypoxic tumors, indicating microbial adaptation to TME changes (Fig. 5A; complete list in Supplementary Table S12). Of the 1,295 identified RNA species, we found 48 significantly different RNA species in the normoxic versus hypoxic tumors in both datasets (39 induced by hypoxia and 9 repressed).

Even with the paucity of annotation in this strain, we found that manual curation identifies adaptive changes in the genes that are primarily involved in metabolism but also several that are involved in oxygen/redox stress, DNA metabolism, and several transcriptional regulators (Fig. 5B). Hypoxic induction of lactate dehydrogenase or citrate synthase may be analogous to what is seen in higher species adapting their metabolism to reduced oxygen environments. The 34 with UniRef90 annotations are listed in Fig. 5C in order of significance. One of the more interesting induced transcriptional regulators is the WhiB family member, which contains a redox-sensitive [4Fe-4S] cluster. The WhiB‐like protein family is exclusively found in *Actinobacteria* and has been reported to be nitric oxide–responsive and oxygen-responsive in other strains (32). This transcriptional regulator may be analogous to the hypoxia-responsive HIF1 transcriptional regulator found in higher species that is largely responsible for transcriptional adaptation to hypoxia. The sum of these findings indicates that intratumoral oxygen tension can both favor the growth of specific microbial populations and elicit adaptive transcriptional responses in microbes that can grow in both normoxic and hypoxic tumors.

## Discussion

Several lines of evidence show that host–microbiome interactions can affect the response to anticancer therapy (14, 33–35). The balance between bacterial and fungal microbes and the metabolites produced by these microbes can affect response to radiotherapy (36, 37). Chronic intestinal inflammation associated with microbial dysbiosis and an intrinsically hypoxic microenvironment creates a niche permissive to microbial colonization of all ranges of partial oxygen pressure (pO_2_) within the tumor. In line with this expectation, we observed that tumor microbiome composition shows distinct patterns as a function of tumor oxygen levels in datasets of patients with colorectal cancer.

Under normal conditions, the intestinal microbiome consists of 1,000+ species of bacteria, most of which are obligate anaerobes (38). Intestinal dysbiosis triggers the rapid expansion of conditional anaerobic pathogens that can disrupt the intestinal mechanical barrier and result in luminal microbes colonizing the tumor. Mean pO_2_ levels within the GI tract range from 42 to 71 mm Hg (7%–10% O_2_) in the colonic muscle wall to 3 mm Hg (0.4% O_2_) in the sigmoid colon, similar to 2.5 mm Hg levels typically observed in colorectal cancer tumors (39, 40). Distribution characteristics of conditional pathogens in both mouse hosts show an overrepresentation of the *Firmicutes* phylum, similar to observations made in human colorectal cancer (41).

Much investigation has gone into the composition of the intratumoral immune system to establish its role in determining the microbes that can colonize a tumor (42). However, there are fewer studies of the metabolic parameters influence the microbes that find a tumor hospitable. The partial pressure of oxygen has been long recognized as an important component of culture media for different microorganisms. Bacteria have been found that range from obligate anaerobes that can only grow in the absence of oxygen to aerobes that grow well in an atmosphere with 21% oxygen. Often, this categorization is due to the presence or absence of enzymes within the microbial genome that are capable of metabolizing toxic oxygen species, such as superoxide dismutase or catalase, which can facilitate growth in an oxidizing environment. Many microbes fall between these extremes and can be categorized as facultative anaerobes or microaerophiles capable of growth in low- or no-oxygen environments. These microbes may be most well adapted for growth in intermediate to low oxygen, which is often found within the TME.

Similarly, although much attention has been given to strains of bacteria that exist within the tumor, more study still needs to be completed on the physiologic state of intratumoral microbes. Microbes could have a very different impact on the tumor due to the expression or silencing of different genes. For example, hypoxic induction of lactate dehydrogenase or citrate synthase in *Cutibacterium* may be analogous to that is seen in higher species adapting their metabolism to reduced oxygen environments. In addition, one of the more interesting induced genes is the transcriptional regulators in the WhiB family, which contains a redox-sensitive (4Fe-4S) cluster. The WhiB‐like protein family is exclusively found in *Actinobacteria* and has been reported to be nitric oxide–responsive and oxygen-responsive in other strains (32). This transcriptional regulator may be analogous to the hypoxia-responsive HIF1 transcriptional regulator found in higher species that is largely responsible for transcriptional adaptation to hypoxia. Our data suggest that adaptation to *Cutibacterium* hypoxia could result in higher levels of microbially produced lactate or citrate. Such metabolites may interact with and change the behavior or morphology of the tumor. For example, tumor lactate has been shown to impact macrophage polarization (43). Such microbe signals have also been shown to impact tumor cell response to radiotherapy (37).

Our study also observed several microbial strains correlated with worse outcomes from radiotherapy in rectal cancer. Notably, we identified *C. glabrata* and *F. canifelinum*, which share close taxonomic similarity to microbes previously related to cancer (*Candida albicans* and *F*. *nucleatum*, respectively). *C. albicans* has been linked to oral squamous cell carcinoma (44) and radioresistant rectal cancer (45) and was shown in a large study to be predictive of metastatic disease across multiple GI tumors (46). Additionally, previous studies have reported that species from the *Fusobacterium* genus are associated with elevated inflammatory response in the colorectal mucosa (47–49). This raises the question of whether the host’s immune response to microbial dysbiosis affects radiotherapy outcomes.

Experimentally, we observed that immunocompromised BALB/c and nude mice hosts show distinct acute immune responses following intratumoral inoculation with several microbial strains found in human colorectal cancer (50). The inoculated mice exhibited mild symptoms of acute immune response associated with changes in tumor growth and response to radiotherapy. We identified that the addition of common commensal bacteria *Lactobacillus acidophilus* and *Streptococcus thermophilus* in the tumor enhances the therapeutic efficacy of local radiotherapy. At the same time, inoculation with *F*. *nucleatum* or microbial coculture, representative of a healthy intestinal microbiome, does not potentiate radiotherapy. These observations support the model that the presence of *Lactobacillus* or *Streptococcus* genera might predict better radiotherapy outcomes in patients with colorectal cancer. However, we note that the 16S-based validation of microbes from additional tumors did not observe either of these microbes. We expect that the well-established differences between the mouse and human gut microbiome could provide a rationale for altered tumor microbiomes (6, 7). In comparison, the presence of *Fusobacterium* predicts worse outcomes {*F. nucleatum* in previous publications [e.g., (8)] and *F. canifelinum* in our study}. Notably, the presence of this organism in tumors has been confirmed by many methods, including 16S-based sequencing in this study.

Perhaps the most intriguing findings come from the metatranscriptomic analysis of conditional pathogen enrichment in hypoxic versus normoxic CT26 colorectal cancer tumors in BALB/c and nude mice. We identified that *Cutibacterium*, a typically anaerobic opportunistic pathogen, most consistently colonizes both the normoxic and hypoxic tumors, displaying broad adaptive flexibility toward different pO_2_ environments. Transcriptomic analysis revealed a systemic reprogramming underlying the adaptive response of the pathogen to varying pO_2_ levels. These results support the model that opportunistic pathogens colonizing colorectal cancer tumors can respond to environmental oxygen levels with adaptive gene expression changes, providing a solid rationale for investigating microbial metabolism in future studies. These results also indicate that it may not be just the presence or absence of a specific microbe within a tumor that dictates its effect on clinical outcomes but the physiologic state of the microbe that can be influenced by the microenvironment in which it resides.

## Supplementary Material

Supplementary Tables Legend Legend for Supplementary Tables

Supplemental Figure S1 Hypoxia scores by stage (A) or sex (B) for the ORIEN radiation-treated cohort.

Supplemental Figure S2 Boxplots comparing immune fractions in low and high hypoxia groups in the ORIEN radiation cohort, displaying cells with significantly different abundance with an adjusted p-value <0.05.

Supplemental Figure S3 Boxplot comparing Fusobacterium counts in low and high hypoxia 16s samples.

Supplemental Figure S4 Boxplots showing the immune cell fractions for immune cells with significantly different expression in high and low hypoxia mouse samples at p-value <0.05. No differences remained significant after correction for multiple hypothesis testing.

Supplementary Data Numbers at risk for the association between low and high hypoxia in COAD and READ tumors (associated with the Kaplan-Meier curve shown in Figure 1D).

Supplementary Data Demographic variables stratified by low and high hypoxia scores, showing no significant differences to be controlled for in the Cox proportional hazards model described in Figure 1D.

Supplementary Data Fold-changes and adjusted p-values for all microbes associated with hypoxic or normoxic tumors in the ORIEN and TCGA datasets (associated with Figure 2A). Results of survival analyses of interactions between microbes and hypoxia in COADREAD patients receiving radiation therapy.

Supplementary Data Kruskal-Walis test results comparing the deconvolved immune cell compositions between hypoxic and normoxic tumors.

Supplementary Data Cox proportional hazards model results for the interaction terms between hypoxia (binary, low or high) with microbe relative abundance in COADREAD tumors treated with radiotherapy.

Supplementary Data Numbers at risk for the association between survival and Fusobacterium canifelinum abundance in COADREAD tumors treated with radiation.

Supplementary Data Differences in the immune cell populations, estimated by RNAseq, within high and low hypoxia mouse tumors, combining the nude and BALB/c mice.

Supplementary Data Differences in the immune cell populations, estimated by RNAseq, within high and low hypoxia mouse tumors, separated by mouse strain.

Supplementary Data Differences in the tumor microbes in low and high hypoxia mouse tumors.

Supplementary Data Selected log2 fold change results for microbes found to be significantly enriched in hypoxic tumors in both mice and human subjects.

Supplementary Data Correlations between tumor microbes enriched in hypoxic tumors in both mice and humans and deconvolved immune cell abundances.

Supplementary Data Log2 fold changes of annotated Cutibacterium genes between hypoxic and normoxic tumors in nude and BALB/c mice.
