# Supplementary material for: The Tumor Microbiome Reacts to Hypoxia and Can Influence Response to Radiation Treatment in Colorectal Cancer
Source: Cancer Res Commun. 2024 Jul 10;4(7):1690–701. doi: 10.1158/2767-9764.CRC-23-0367 (PMC11234499; doi:10.1158/2767-9764.CRC-23-0367)
Supplement: Supplementary Tables Legend — Legend for Supplementary Tables [file crc-23-0367_supplementary_tables_legend_suppsl.docx]

# SUPPLEMENTARY TABLES LEGEND

**Table S1.** Numbers at risk for the association between low and high hypoxia in COAD and READ tumors (associated with the Kaplan-Meier curve shown in **Figure 1D**).

**Table S2.** Demographic variables stratified by low and high hypoxia scores, showing no significant differences to be controlled for in the Cox proportional hazards model described in **Figure 1D**.

**Table S3.** Fold-changes and adjusted p-values for all microbes associated with hypoxic or normoxic tumors in the ORIEN and TCGA datasets (associated with **Figure 2A**).

Results of survival analyses of interactions between microbes and hypoxia in COADREAD patients receiving radiation therapy

**Table S4.** Kruskal-Walis test results comparing the deconvolved immune cell compositions between hypoxic and normoxic tumors.

**Table S5.** Cox proportional hazards model results for the interaction terms between hypoxia (binary, low or high) with microbe relative abundance in COADREAD tumors treated with radiotherapy.

**Table S6.** Numbers at risk for the association between survival and *Fusobacterium canifelinum* abundance in COADREAD tumors treated with radiation.

**Table S7.** Differences in the immune cell populations, estimated by RNAseq, within high and low hypoxia mouse tumors, combining the nude and BALB/c mice.

**Table S8.** Differences in the immune cell populations, estimated by RNAseq, within high and low hypoxia mouse tumors, separated by mouse strain.

**Table S9.** Differences in the tumor microbes in low and high hypoxia mouse tumors.

**Table S10.** Selected log2 fold change results for microbes found to be significantly enriched in hypoxic tumors in both mice and human subjects.

**Table S11.** Correlations between tumor microbes enriched in hypoxic tumors in both mice and humans and deconvolved immune cell abundances.

**Table S12.** Log2 fold changes of annotated *Cutibacterium* genes between hypoxic and normoxic tumors in nude and BALB/c mice
