# Supplementary figures and images for: The Tumor Microbiome Reacts to Hypoxia and Can Influence Response to Radiation Treatment in Colorectal Cancer
Source: Cancer Res Commun. 2024 Jul 10;4(7):1690–701. doi: 10.1158/2767-9764.CRC-23-0367 (PMC11234499; doi:10.1158/2767-9764.CRC-23-0367)

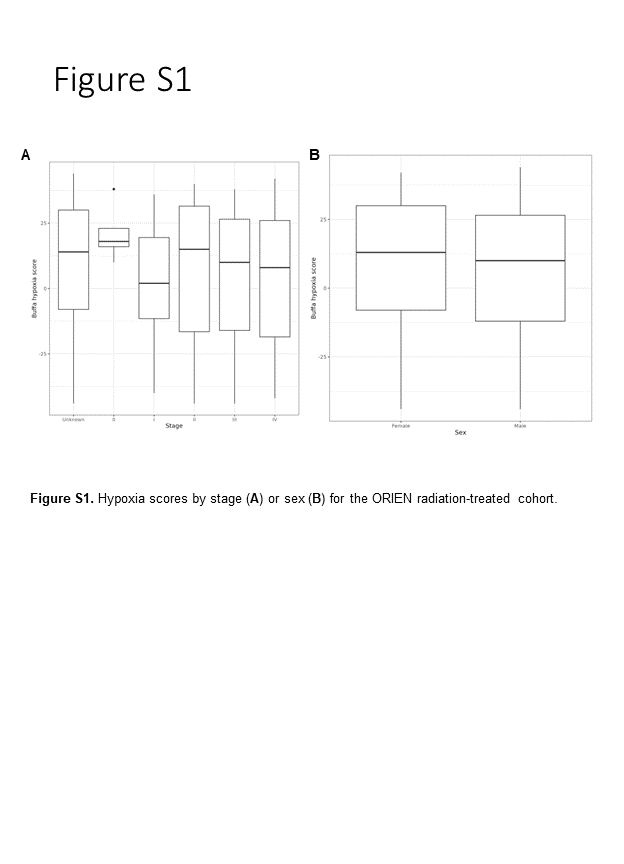

Supplement: Supplemental Figure S1 — Hypoxia scores by stage (A) or sex (B) for the ORIEN radiation-treated cohort. [file crc-23-0367_supplemental_figure_s1_supps1.png]

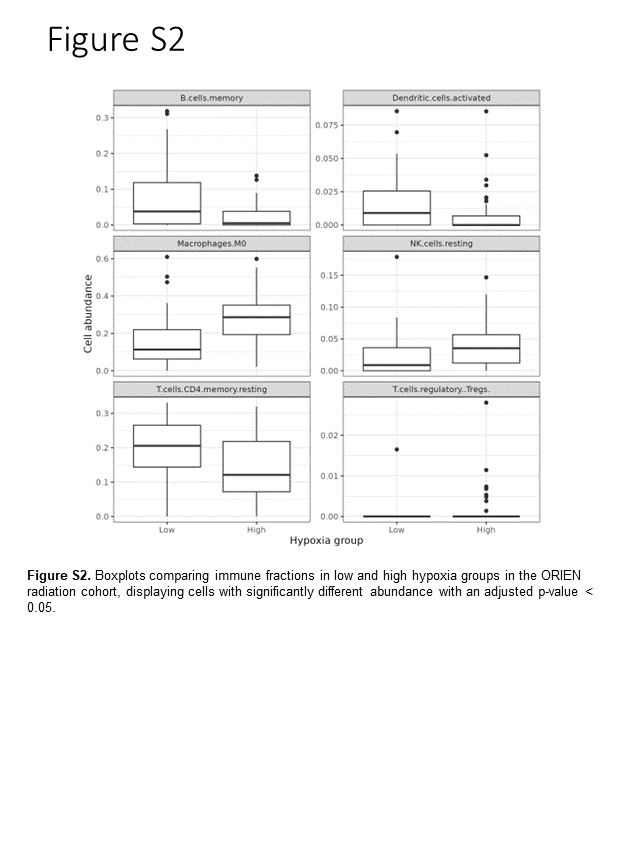

Supplement: Supplemental Figure S2 — Boxplots comparing immune fractions in low and high hypoxia groups in the ORIEN radiation cohort, displaying cells with significantly different abundance with an adjusted p-value <0.05. [file crc-23-0367_supplemental_figure_s2_supps2.png]

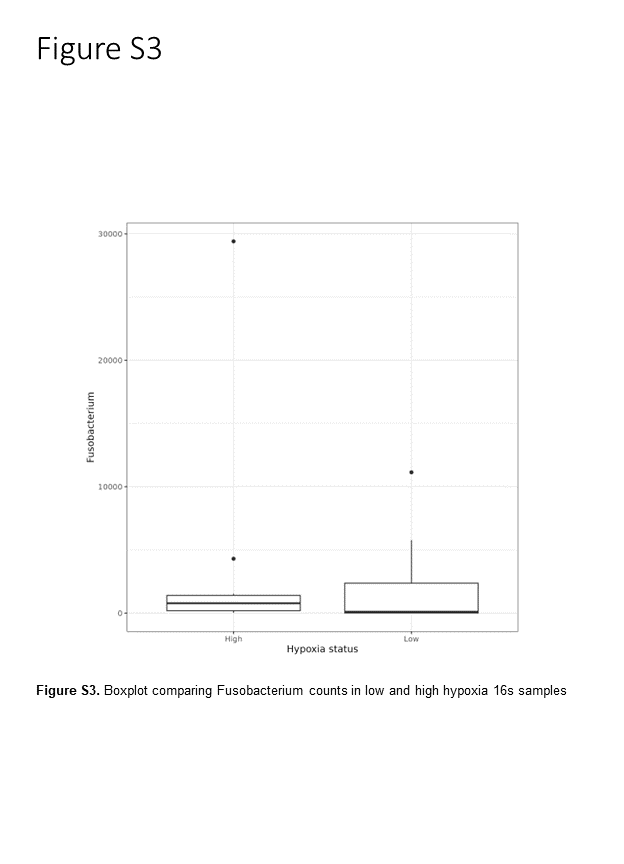

Supplement: Supplemental Figure S3 — Boxplot comparing Fusobacterium counts in low and high hypoxia 16s samples. [file crc-23-0367_supplemental_figure_s3_supps3.png]

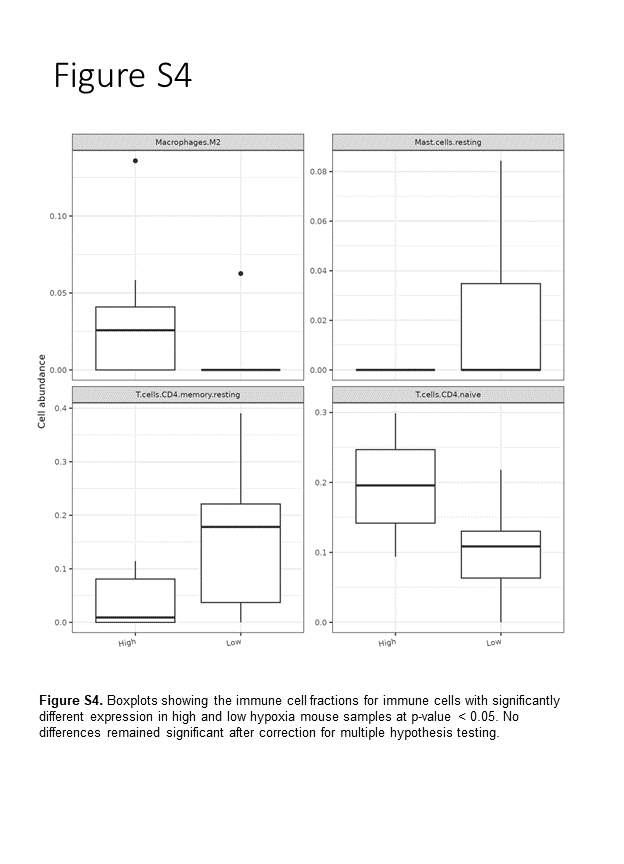

Supplement: Supplemental Figure S4 — Boxplots showing the immune cell fractions for immune cells with significantly different expression in high and low hypoxia mouse samples at p-value <0.05. No differences remained significant after correction for multiple hypothesis testing. [file crc-23-0367_supplemental_figure_s4_supps4.png]
